# X-ray scattering based scanning tomography for imaging and structural characterization of cellulose in plants

**DOI:** 10.1107/S1600577524004387

**Published:** 2024-06-25

**Authors:** Lin Yang

**Affiliations:** a National Synchrotron Light Source II Brookhaven National Laboratory Bldg 745 Upton NY11973 USA; University of Malaga, Spain

**Keywords:** X-ray scattering, imaging, tomography, plant cellulose

## Abstract

X-ray and neutron scattering have long been used for structural characterization of cellulose in plants. Due to averaging over the illuminated sample volume, these measurements traditionally overlooked the compositional and morphological heterogeneity within the sample. Here, a scanning tomographic imaging method is described, using contrast derived from the X-ray scattering intensity, for virtually sectioning the sample to reveal its internal structure at a resolution of a few micrometres. This method provides a means for retrieving the local scattering signal that corresponds to any voxel within the virtual section, enabling characterization of the local structure using traditional data-analysis methods. This is accomplished through tomographic reconstruction of the spatial distribution of a handful of mathematical components identified by non-negative matrix factorization from the large dataset of X-ray scattering intensity. Joint analysis of multiple datasets, to find similarity between voxels by clustering of the decomposed data, could help elucidate systematic differences between samples, such as those expected from genetic modifications, chemical treatments or fungal decay. The spatial distribution of the microfibril angle can also be analyzed, based on the tomographically reconstructed scattering intensity as a function of the azimuthal angle.

## Abstract 2

Tomographic virtual sectioning of plant samples based on X-ray scattering intensity provides spatially resolved characterization of the structure and organization of cellulose in plants.

## 1. Introduction

X-ray and neutron scattering patterns from plant cell walls typically consist of diffraction peaks from crystalline cellulose and diffuse contributions from amorphous components like lignin. The scattering data can be used to characterize the abundance and degree of organization of cellulose structures, based on derived quantities such as the crystallinity index (CI) (Thygesen *et al.*, 2005 ▸) and the degrees of correlation between cellulose fibrils. Scattering methods therefore have been used in studies of breakdown of biomass feedstock for bioenergy production (Dadi *et al.*, 2006 ▸; Samayam *et al.*, 2011 ▸; Inouye *et al.*, 2014 ▸) and natural fungal decay of wood (Castaño *et al.*, 2022 ▸; Floudas *et al.*, 2022 ▸). Spatially resolved measurements using micro-focused X-ray beams can reveal the structural heterogeneity in the sample and therefore have the potential to provide a more precise description of how these processes take place. For instance, scanning mapping of thin sections has been used to quantify the local orientation of the cellulose microfibrils in secondary cell walls (Lichtenegger *et al.*, 1999 ▸). However, preparing sections that are cut perpendicular to the growth direction and therefore reveal the cell-wall architecture is not always feasible, especially for samples that have been chemically treated or have already decayed naturally. Furthermore, due to the fiber geometry of the cellulose structures, the observed scattering intensity can be highly dependent on local orientation of the cellulose fibrils, making data analysis difficult. Here, we use scanning scattering tomography to circumvent both issues.

**Figure 1:**
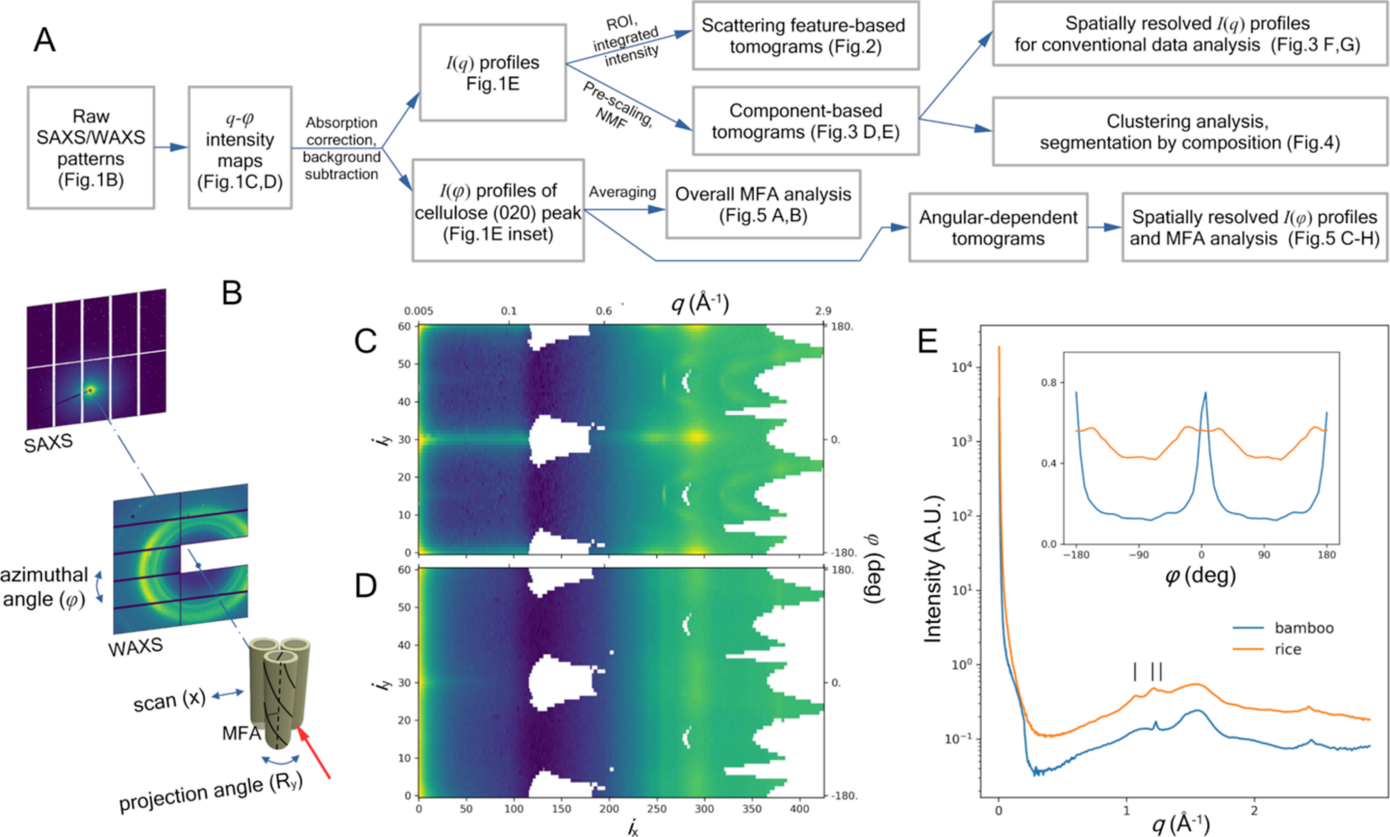
The overall data-processing workflow and representative data collected in scanning tomography measurements. (*A*) An overview of all the processing and analysis described in this article. The experimental geometry and typical scattering patterns are shown in (*B*). The plant sample is schematically depicted as a collection of hollow cylinders that represent the cell walls. The spiral lines indicate the orientation of cellulose fibrils with a non-zero MFA value. The sample is scanned across the X-ray beam (red arrow) at a series of projection angles during measurements. The scattering patterns were collected on two separate detectors, for small- and wide-angle X-ray scattering (SAXS and WAXS), and then reformatted and merged into *q*–φ maps. The relevant data collection and processing details have been described elsewhere (Yang *et al.*, 2022 ▸). The data shown are from (*C*) a bamboo sample and (*D*) a rice sample, respectively. The lower horizontal (*i*_*x*_) and left vertical axes (*i*_*y*_) are the pixel indices of the intensity map. The intensity profiles averaged over all azimuthal angles, φ, are shown in (*E*), while the angular intensity distributions near the maximum of the cellulose peak are shown in the inset. The scattering intensity spans a dynamic range of several orders of magnitudes. Therefore, in (*C*) and (*D*), the scattering intensity has been multiplied with a factor of *q*^2^ so that the features at both high *q* and low *q* are visible with the same color scale. Similarly, a non-uniform *q* grid is used to show features more clearly at both high *q* and low *q*. The black vertical lines in (*E*) indicate the peaks that are attributed to the structure of starch.

Performing tomographic imaging based on X-ray scattering contrast has been applied to polymer materials (Stribeck *et al.*, 2006 ▸; Schroer *et al.*, 2006 ▸) as well as biological tissues (Jensen *et al.*, 2011 ▸; Liebi *et al.*, 2015 ▸; Schaff *et al.*, 2015 ▸; Georgiadis *et al.*, 2021 ▸; De Falco *et al.*, 2021 ▸). In general, the observed scattering signals may be dependent on the orientation of the underlying structure (*e.g.* collagen and minerals in bones, or myelin in nerve tissues). The tomographic reconstruction algorithm therefore needs to account for this projection-angle dependence. On the other hand, in cases where the basis for the reconstruction is invariant during sample rotation (see discussion by De Falco *et al.*, 2021 ▸), the reconstruction can be much simplified. Cellulose in plant cell walls is the primary source of the observed X-ray scattering intensity. The scattering from an isolated cellulose fibril is rotationally invariant with respect to the fiber axis. Plant cells in tissues that are most abundant in cellulose are cylindrical structures that are elongated along the growth direction. The overall scattering from the enclosed cell walls resembles that from cellulose fibrils, with the growth direction being the average cellulose fiber direction. However, the presence of the microfibril angle (MFA), which is the angle between the fiber axis and the growth direction [see Fig. 1 ▸(*B*)], gives rise to a split peak along the azimuthal angular direction [*e.g.* Fig. 1 ▸(*D*) and the inset of Fig. 1 ▸(*E*)]. Nevertheless, the scattering intensity integrated over all azimuthal angles does not depend on sample rotation about the growth direction. Therefore, as long as the plant sample (*e.g.* a matchstick cut from wood, or the stem of a small plant) is positioned such that the growth direction coincides with the rotation axis of the projection angle and is perpendicular to the incident beam, the rotational invariance holds. The reconstruction can then be performed using existing tools developed for tomographic imaging based on observables that are scalers, such as X-ray absorption or fluorescence emission.

Collecting the scattering data as described above can also be helpful in determining the MFA from the observed intensity. Typically, the cell-wall geometry must be separately characterized to define the cell-wall orientation as an input parameter when calculating the MFA distribution (Cave, 1997 ▸; Rüggeberg *et al.*, 2013 ▸). In tomographic data collection, the sample is rotated for observation from different projection angles. In the scattering intensity averaged over all projection angles, all cell-wall orientations contribute equally. The overall MFA distribution can therefore be evaluated based on the scattering data alone. In addition, under the assumption of rotational invariance of the angular scattering intensity profile, we can further retrieve the local MFA and visualize its spatial distribution.

We demonstrate the workflow of scanning scattering tomography using, as examples, a bamboo sample cut from the internode and the intact stalk from several rice plants.

## 2. Tomography based on the intensity of specific features in the scattering data

Fig. 1 ▸ shows typical X-ray scattering patterns from a bamboo sample and a rice sample. As part of the uniform data-processing workflow summarized in Fig. 1 ▸(*A*), we first convert the X-ray scattering data into intensity maps with coordinates of the scattering vector *q* [defined as 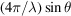, where λ is the X-ray wavelength and 2θ is the scattering angle] and the azimuthal angle φ. The most prominent feature in a typical scattering pattern is the well documented diffraction peaks from crystalline cellulose. There is also a diffuse peak at ∼0.1 Å^−1^ that is perpendicular to the fiber axis, which can be well explained by the correlation between cellulose microfibrils (Jakob *et al.*, 1994 ▸; Kennedy *et al.*, 2007 ▸; Fernandes *et al.*, 2011 ▸; Penttilä *et al.*, 2019 ▸). Finally, the streak at extremely low *q* has been attributed to the porous structure of plant tissue (Jakob *et al.*, 1996 ▸; Nishiyama *et al.*, 2014 ▸).

Crystalline cellulose and amorphous structural components (*e.g.* lignin) contribute to this intensity map differently. In the context of tomographic reconstruction, we consider the contribution from a volume element in the virtual cross section during sample rotation. The scattering from amorphous components is isotropic. In contrast, for crystalline cellulose, the scattering intensity is a function of the azimuthal angle, and dependent on the microfibril orientation in the cell walls (Cave, 1997 ▸; Barnett & Bonham, 2004 ▸). We have two different options for analyzing these data. A unsplit cellulose peak along the φ axis in the intensity map that is independent of the projection angle during tomographic data collection, as is the case for the bamboo sample [see the inset of Fig. 1 ▸(*E*)], suggests that most cellulose fibrils are highly oriented with a near-zero MFA. We could then focus on the intensity profile at φ = 0 (on the equator in the fiber-diffraction diagram): *I*_e_(*q*) = *I*(*q*, φ = 0). This assures that the intensity is free of contamination from the cellulose peaks outside of the zeroth layer line. More generally, however, the MFA is not a single value but a distribution. Multiple structural components may also have different orientational distributions [*e.g.* cellulose and starch contributions in Fig. 1 ▸(*D*), as described below]. It is therefore preferred to work with the intensity integrated over all azimuthal angles: 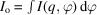, which is equivalent to the data from powder diffraction measurements (commonly referred to as XRD in the literature). This is because the integral over φ negates the MFA-induced intensity redistribution at the fibril level. Therefore, *I*_o_(*q*) is independent of the projection angle, even if the φ-dependent profile of the contribution from the individual cells could vary with the projection angle due to the cell-wall architecture.

With the rotational invariance of the scattering intensity established, we can use general-purpose software like *tomopy* (Gürsoy *et al.*, 2014 ▸) for tomographic reconstruction. The contribution to the overall scattering intensity from cell walls that are not aligned with the growth direction is expected to be small and therefore neglected. As we will see in the following, this assumption does not seem to affect tomographic reconstruction and therefore likely holds true for the examples below.

**Figure 2:**
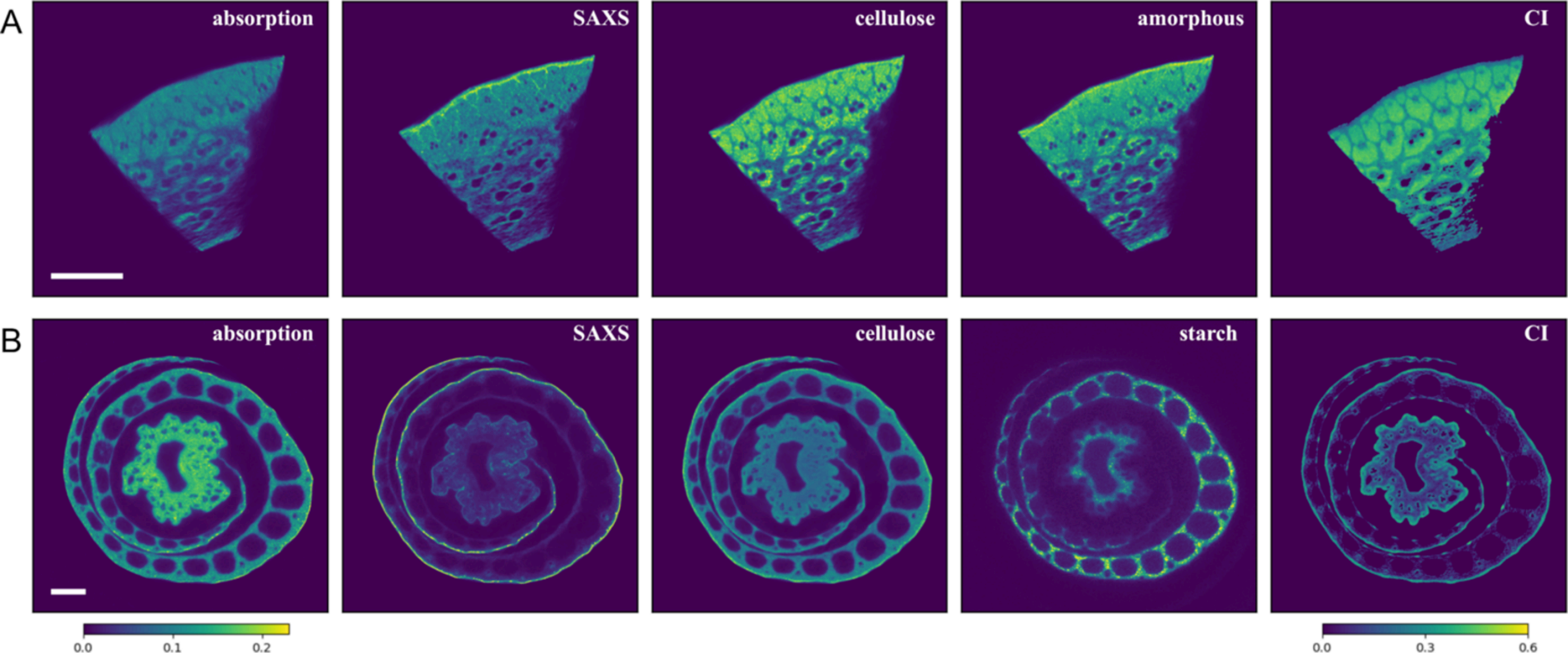
Tomograms based on features extracted from the X-ray scattering data for the (*A*) bamboo and (*B*) rice samples. The scale bars represent 0.5 mm. The absorption-based tomograms are included for reference. The scattering intensity based tomograms were obtained as described in Section 5. The color bars are for the absorption and CI maps only. All the other maps show relative values and follow the same color code.

As a proof of concept, we have previously demonstrated (Yang *et al.*, 2022 ▸) tomographic sectioning of a poplar stem based on the intensity of the cellulose (020) peak in the scattering data. Fig. 2 ▸ shows similar results for the bamboo and rice samples. In principle, any parameter extracted from conventional analysis of the individual scattering patterns can be used for tomographic reconstruction, so long as they are additive and therefore follow the Radon transform. For instance, rice plants store starch in granules in the leaf sheath and culm during vegetative growth (Perez *et al.*, 1971 ▸; Sato, 1984 ▸). Diffraction peaks that are characteristic of the A-type starch structure at *q* ≃ 1.07, 1.21 and 1.27 Å^−1^ (scattering angles of 2θ = 15.2, 17.2 and 18.0° at 8 keV, respectively; Kadan & Pepperman, 2002 ▸) are visible in the data shown in Fig. 1 ▸(*C*). Rice plants are also known to contain silica. However, no clear signature from silica is visible in the data, likely due to its amorphous form. We can use the intensity of the most prominent peaks that correspond to cellulose and starch in the scattering data (see details in Section 5.4) to estimate their spatial distributions based on tomographic reconstruction. This is demonstrated in Fig. 2 ▸(*B*). Notably, while the starch distribution appears uniform in the leaf sheath, in the culm there is a clear radial distribution, with higher concentration on the inner periphery. Starch also appears absent in tissues that show high values in the CI map, suggesting those specific cell types are not involved in starch storage.

The intensity-based tomograms are direct measures of material abundance (cellulose and starch in this example). However, the numerical values are not calibrated. They may need to be scaled to account for the difference in the integrating *q* ranges used for preparing the sinograms when making comparisons between datasets (different samples or plant individuals). The values in the absorption tomogram correspond to the logarithm of absorption per voxel and are therefore comparable between tomograms. Indirect calculations of other quantities are possible. In particular, the cellulose crystallinity can be calculated based on the intensity tomograms for the cellulose (020) peak, and for the intensity minimum between the (020) and (110) peaks, as shown in the CI maps in Fig. 2 ▸. While the numerical value of the CI cannot be taken at face value, as has been discussed in the literature (*e.g.* French & Cintrón, 2013 ▸; Lindner *et al.*, 2015 ▸), it is adequate for quantitative comparisons.

The small-angle X-ray scattering (SAXS) tomograms correspond to the scattering intensity in the *q* range where the cellulose-fibril correlation peak is expected to appear. Correlations between fibrils can affect both the magnitude and the position of this peak. Therefore, the numerical values in these maps cannot be simplistically interpreted as the abundance of a structural species. However, it could serve as a starting point to identify regions for further inspection, for instance by using the method for extracting local scattering intensity described below.

## 3. Recovering spatially resolved X-ray scattering patterns based on component decomposition and segmentation of scattering tomogram by composition

While the tomograms presented in the previous section provide intuitive visualization of material distribution, spatially resolved scattering data that correspond to each individual voxel in the tomogram would permit evaluation of the local structure based on traditional X-ray scattering analysis methods. In principle, tomographic reconstruction can be performed for each *q* value in the scattering data, to recover the local scattering intensity, as has been previously demonstrated (Jensen *et al.*, 2011 ▸; Birkbak *et al.*, 2015 ▸). Here, we take a different approach that is computationally less costly, by reducing the number of required reconstruction calculations from a few hundred (>400 *q* points in our data) to under ten, and potentially more reliable since limiting the number of components assures that the reconstructions are minimally affected by the statistical noise in the low-intensity portions (*q* ≃ 0.3 Å^−1^) of the scattering data (see Fig. S1 of the supporting information). Representing the data with a small number of components amounts to reducing the dimensionality of the dataset and facilitates the application of machine-learning methods like clustering analysis, as we will show below.

**Figure 3:**
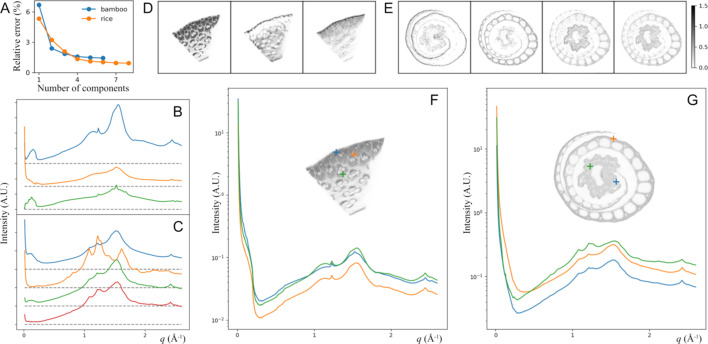
Spatially resolved scattering intensity derived from the tomography data. For each sample, the entire dataset can be decomposed using a basis set, for which the number of vectors is determined based on the relative residue error [(*A*), see Section 5 for details]. (*B*) and (*C*) show the three basis vectors chosen for bamboo and the four vectors chosen for rice, respectively, where the vectors are offset for clarity and the dashed lines indicate the baselines (zero intensity). Decomposing the scattering data using these basis sets gives the sinograms for each basis vector, which are then converted into spatial distributions by tomographic reconstruction, shown in (*D*) and (*E*), where the color bar shows the relative magnitude. The scattering intensity for any given position in the virtual cross section can then be reconstructed, based on the local concentration of each component. This is shown in (*F*) and (*G*), with the scattering profiles corresponding to the three locations indicated in the insets by plus signs with the same color code.

It is reasonable to assume that the sample being measured contains only a finite number of structural components. If we could identify the scattering profiles that correspond to these components, by decomposing the scattering data into this basis set, we would be able to quantify the contribution from each component in a spatially resolved manner. We could then use tomographic reconstruction to convert these distributions as functions of angle and position to the cross-sectional distributions of the same components. In practice, the number of components and their corresponding scattering profiles are typically unknown. However, we can decompose the scattering patterns based on a set of mathematically selected components, or basis vectors, and similarly recover the local scattering intensity as we would do for physical components. Fig. 3 ▸ shows this process being applied to the bamboo and rice samples, once we decompose the scattering profile into three and four components, respectively, using non-negative matrix factorization (NMF, see Section 5.6 for details). As examples, the reconstructed local scattering profiles are shown for three different locations in each sample.

These results are in general consistent with the tomograms in Fig. 2 ▸. For instance, for the rice sample, the top two basis vectors in Fig. 3 ▸(*C*) exhibit the scattering characteristics from the cellulose fibril correlation and starch. Not surprisingly, the resulting tomograms are similar to the SAXS and starch maps in Fig. 2 ▸(*B*). On the other hand, the NMF analysis highlights some features in the scattering data that may be easily missed unless being specifically looked for. For the bamboo samples, the third basis vector shows several sharp but low-intensity peaks. We are unable to identify the corresponding structural component. Since this component is abundant in the cortex (near the outer surface of the culm), it could be partially due to water-insoluble components that typically need to be removed when bamboo is used as a structural material (Nkeuwa *et al.*, 2022 ▸). Bamboo is also known to store starch in the undifferentiated cells between vascular bundles (Wang *et al.*, 2016 ▸). The extracted starch has been identified to have the A-type structure (Felisberto *et al.*, 2020 ▸). However, since the presence of starch is seasonal, transient structural species that form during starch metabolism could have contributed to what we observe as well.

**Figure 4:**
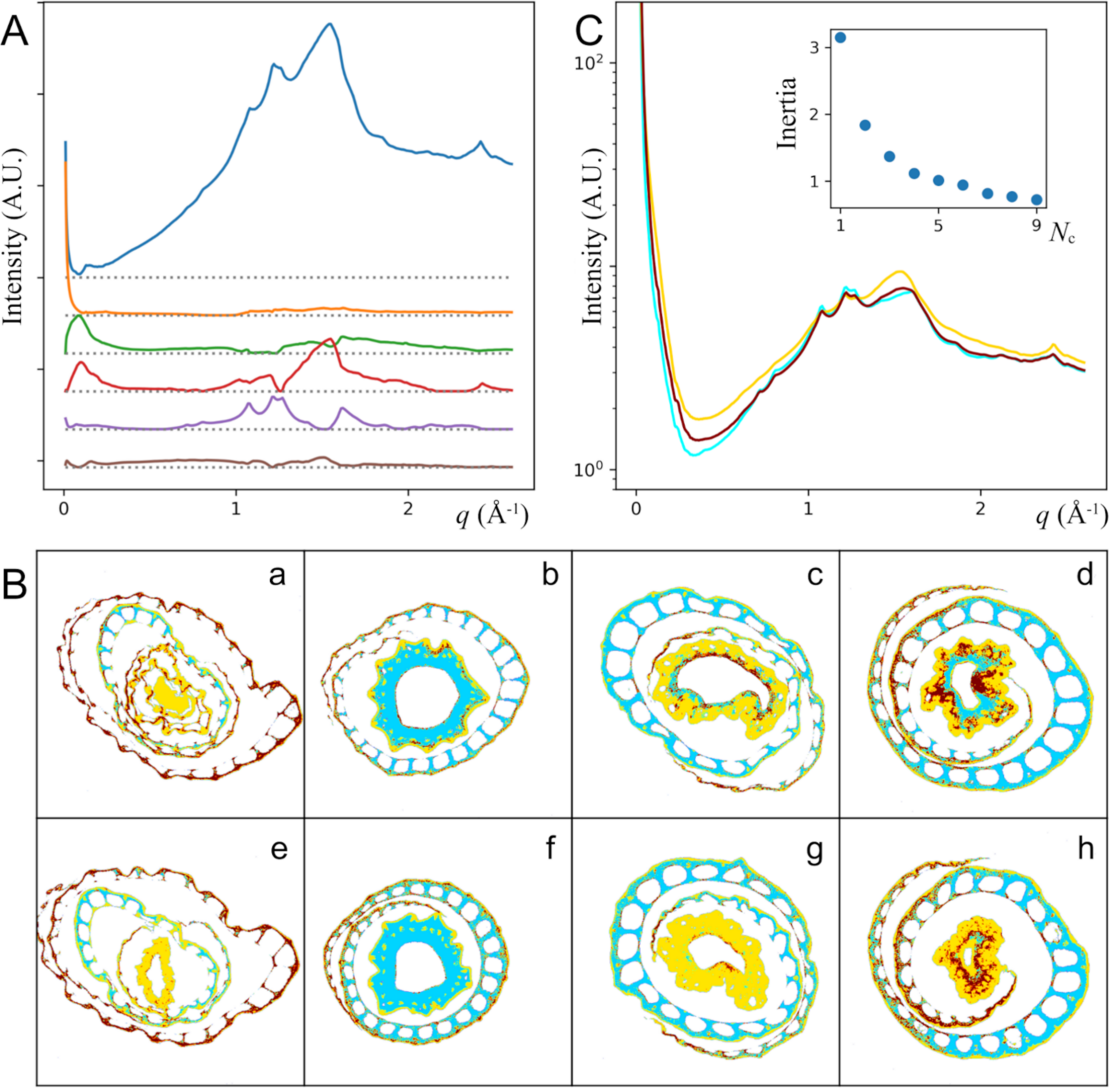
(*A*) Six components are used to decompose the data from four individual rice plants [columns in (*B*)], collected at two different positions [rows in (*B*)] along the growth direction. The voxels in the tomograms (*B*) have been grouped into three clusters, represented by different colors, based on the relative magnitude of the six components as described in the main text. The average scattering intensity profile that corresponds to each cluster is shown in (*C*), using the same color code as in (*B*). The inset shows the inertia of the *k*-means analysis as a function of the chosen number of clusters. The four rice plants correspond to the genotypes MOMT4 (*a*, *e*), MOMT9 (*b*, *f*), WT (*c*, *g*, wildtype) and VC (*d*, *h*, empty vector control) (Dwivedi *et al.*, 2024 ▸).

The components identified by NMF reflect prominent features observed in the scattering data, but in general they do not correspond to physical constituents. The NMF algorithm only assures that the basis vectors have positive values. Some vectors may contain features that are not realistic in scattering data, such as a dip in intensity that corresponds to the inverse of a peak from a different basis vector [see examples in Fig. 4 ▸(*A*)]. NMF results are not unique, but rather depend on the input parameters. NMF also does not account for any feature that varies slightly in position (*e.g.* the fibril correlation peak) in the dataset as a single component, but rather interprets that as a superposition of different peaks. These are well known issues and an active research area in the X-ray scattering and powder diffraction community (*e.g.* Maffettone *et al.*, 2021 ▸). Nevertheless, the recomposed spatially resolved scattering data are expected to be independent of the basis set used for the decomposition, as long as the process is sufficiently accurate in describing the original experimental data. Even when the tomographic reconstruction is marginal [*e.g.* the second component for the bamboo sample, as shown in Fig. 3 ▸(*C*)], the salient features in the scattering pattern are still captured.

The recomposed scattering data can now be analyzed using methods developed for conventional scattering data. The large number of data points (∼10^5^ per sample in these examples) makes it very computationally costly to perform analysis (*e.g.* model fitting) on the individual scattering intensity profiles before tomographic reconstruction. This is also true for the recomposed data. In cases where features in the NMF components can all be accounted for by physical constituents, the individual components can be analyzed first. For instance, they can be fitted to find the intensity of known crystalline cellulose peaks. These results can then be used either to construct tomograms as discussed in Section 2 or to derive the results for the same analysis for the recomposed per-voxel data.

In samples with complex compositions, it may be more important to understand how the overall composition varies spatially, rather than to identify the distribution of each pure component. This is analogous to the CI that captures the relative abundance of crystalline cellulose and the amorphous component being more informative than the abundance of either the crystalline or amorphous component alone. For this purpose, decomposition by NMF can be seen as a process of reducing the dimensionality of the parameter space, to provide inputs to further composition analysis, for instance by clustering. This is shown in Fig. 4 ▸. Here, we have performed NMF to decompose eight sets of scattering data from samples of four genotypes into six common components, followed by a clustering analysis using *k*-means to ‘segment’ the virtual section into three clusters, shown as different colors. The scattering intensities representative of these clusters are shown in Fig. 4 ▸(*C*).

This analysis is helpful when the NMF components do not show features that can be clearly attributed to structural components and therefore cannot be interpreted as scattering intensity from physical structures. In contrast, the cluster averages do represent actual scattering intensity and are therefore interpretable. In this specific example, the main difference between the clusters appears to be starch abundance, with the gold component being the most cellulose rich and the cyan component being the most starch rich. The biological study (Dwivedi *et al.*, 2024 ▸) that produced these rice plants was designed to elucidate the consequence of introducing genes that affect the production of lignin, which is unclear from this analysis due to the presence of starch. On the other hand, the distribution of these components clearly varies between plants and between different locations within the same plant. Interpretation of these data would be more informative with better defined biological context.

## 4. MFA analysis

**Figure 5:**
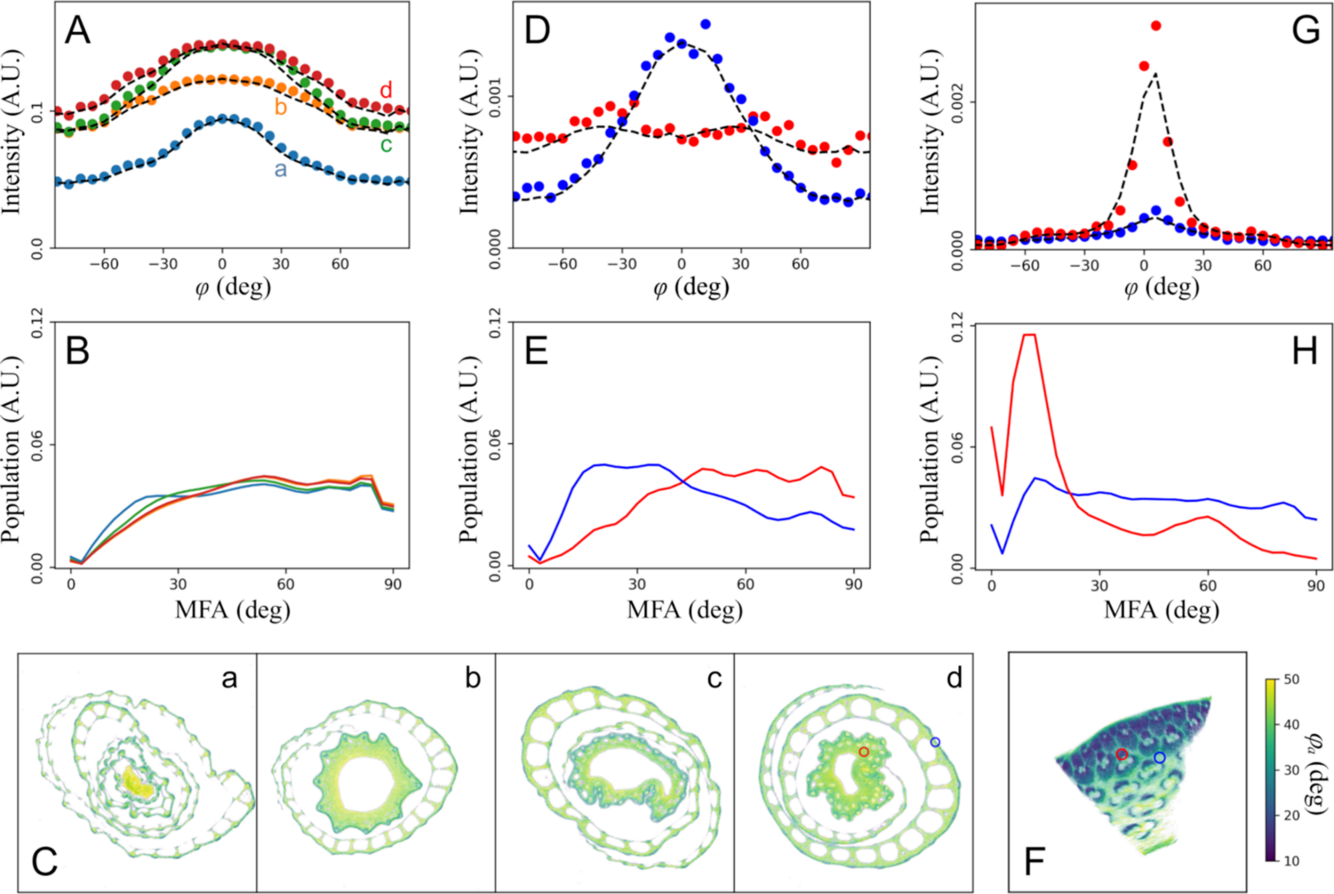
(*A*) The azimuthal angular intensity profiles of the crystalline cellulose peaks corresponding to the four samples shown in Fig. 4 ▸. The calculated MFA distributions are shown in (*B*), using the same color code. The calculated intensity profiles are shown as dashed lines in (*A*). The overall distribution of MFA is shown in (*C*), represented by φ_a_ as defined in the main text. The color in these maps corresponds to the angular value, while the transparency is based on the sample absorption, to avoid confusing φ_a_ values where the scattering intensity is low. The angular dependence of each voxel in the virtual section can be extracted and analyzed using the same method as the averaged data. This is shown for two locations (colored circles) for sample *d* as examples, with the local angular intensity profiles shown in (*D*) and the MFA distributions shown in (*E*), using the same color code. A similar analysis has been carried out in (*F*)–(*H*) for the bamboo sample for comparison.

As discussed in the *Introduction*[Sec sec1], averaging the scattering data over all projection angles eliminates the contribution from cell-wall architecture in the observed azimuthal angle dependence in the scattering intensity. The MFA distribution can then be estimated based on a finite number of discrete MFA values, similar to the method described by Rüggeberg *et al.* (2013 ▸). To do so, the contribution from amorphous components is first subtracted, assuming that it can be represented by the scattering intensity just outside of the crystalline cellulose peak (*q* = 1.8–1.9 Å^−1^), and the contribution from higher-order cellulose peaks is ignored. These approximations will likely introduce some inaccuracies in the subsequent MFA analysis, therefore we have employed a simplified method (see Section 5) for extracting the approximate MFA distribution, instead of fitting the data using an assumed functional form for the distribution. Figs. 5 ▸(*A*) and 5 ▸(*B*) show this analysis being applied to the rice plants from Fig. 4 ▸.

Assuming that the azimuthal angle dependence of the intensity from each voxel is invariant with respect to the projection angle, we can perform tomographic reconstruction for each azimuthal angular position (φ) and retrieve the azimuthal angular intensity profile for each voxel, *I*(φ; *x*, *y*). This assumption would hold true if the distribution of cell-wall orientation within each voxel is close to isotropic, which is more likely when the voxel size is large (∼5 µm in this study) compared with the cell size. The sinograms for these angular positions do not exhibit any anomalies (*e.g.* intensity discontinuity or usually symmetry) and the reconstructions show the same morphology as other tomograms from the same sample (see the supporting movie in the supporting information). Therefore, at a minimum, these tomograms can be considered as a reasonable approximation of the ground truth. However, due to the underlying assumption of invariance in the azimuthal intensity profile, they should be considered qualitative until more rigorous validation can be performed.

To visualize the MFA distribution, we plot [Figs. 5 ▸(*C*) and 5(*F*)] the nominal MFA value, defined as the intensity-weighted average of the absolute value of the azimuthal angular positions for each reconstructed scattering profile:
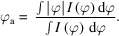
This is not a direct measurement of the MFA. But small MFAs do lead to a low φ_a_ value. In the extreme case, φ_a_ = 0 if all microfibrils are aligned with the growth direction. There is clearly a variation of MFA within these plants. In particular, the sclerenchyma cells in the periphery of the leaf sheath and the exterior surface of the culm exhibit the lowest values of MFAs (see the supporting movie). This is qualitatively consistent with the azimuthal intensity profiles observed during data collection, *e.g.* the angular distribution is narrower when the beam illuminates the exterior of the sample compared with when the beam path traverses the center of the stalk (Fig. S8). With the reconstructed full azimuthal angular intensity profile, we can also perform the MFA analysis for each voxel in the virtual section, as shown in Figs. 5 ▸(*D*) and 5 ▸(*E*).

This analysis is also performed for the bamboo sample for comparison, with the effective MFA shown in Fig. 5 ▸(*F*) and the local MFA analysis shown in Figs. 5 ▸(*G*) and 5 ▸(*H*). The MFA distributions in the fibers and parenchyma are consistent with the results reported in a previous study (Ahvenainen *et al.*, 2017 ▸), where data carefully collected from each type of tissues were analyzed based on cell-wall architecture obtained from absorption-based X-ray microtomography.

## 5. Experimental and data-analysis details

The overall workflow for data processing and analysis is summarized in Fig. 1 ▸(*A*). Detailed descriptions of each step are given below. This workflow has been implemented in the *lixtools* Python package (https://github.com/NSLS-II-LIX/lixtools).

### 5.1. Samples

The bamboo sample, possibly *Phyllostachys bissetii*, was harvested from a local residential area. An ∼20 mm-long section was extracted from the internode of a plant that was ∼10 mm in outer diameter. The rice samples were provided by Dr Chang-Jun Liu’s group (Dwivedi *et al.*, 2024 ▸). Four different samples (see Figs. 4 ▸ and 5 ▸) were measured, including two MOMT variants in which lignin biosynthesis had been modified. All samples were air dried and measured without any further treatment.

### 5.2. Data collection

The X-ray scattering experiments were performed at the Life Science X-ray Scattering (LiX) beamline (Yang *et al.*, 2022 ▸) at the NSLS-II synchrotron source of Brookhaven National Laboratory (Upton, New York, USA). The X-ray beam was focused to the sample position within a spot size of ∼5 µm. The X-ray energy was 15 keV. Scattering patterns were collected at a frame rate of 20 Hz, as the sample was scanned along the *x* axis (perpendicular to the beam and the growth direction) in fly scanning mode. That is, the motion controller (Newport XPS) produced pulses at a series of predetermined positions to trigger the detectors as the sample stage moved continuously. The incident and transmitted beam intensities were also recorded as previously described (Yang *et al.*, 2020 ▸, 2022 ▸). The averaged sample position during the detector exposures followed 5 µm steps. A total of 121 projection angles (*R*_*y*_ at 1.5° intervals) were collected, divided into eight groups of evenly spaced angles, such that each group covered half a rotation, *e.g.* 0, 5, 10,…, 180°, followed by 1.5, 6.5, 11.6,…, 176.5°, *etc*. Between groups, the sample was shifted slightly (10 µm) along the rotation axis to limit radiation damage. This implicitly assumes that the structure does not change significantly along the growth direction. This is confirmed by the general lack of discontinuities in the sinograms that would otherwise arise from structural differences that are adjacent in space but measured far apart in projection angles (the beginning of an angular group and the end of the next one, *e.g.* 0 and 176.5°). Since these angular positions are also measured far apart in time, structural changes due to radiation damage would produce similar discontinuities. Sinograms, especially those based on mathematical components (see Fig. S9), can therefore be used effectively to monitor radiation damage.

### 5.3. Assembling data for analysis

For each data point (*x* and *R*_*y*_), the X-ray scattering patterns from the two detectors were merged into a combined *q*–φ map, with central symmetry applied to increase the detector coverage in the reciprocal space (Yang *et al.*, 2022 ▸). This intensity map was then further reduced to one-dimensional *q* and φ profiles. The background scattering, for which we take the average intensity from the scattering patterns of the lowest overall intensity when the sample is not illuminated by the beam, is subtracted based on the transmitted beam intensity. In this process, the data are also corrected by sample absorption, as measured by the incident and transmitted intensities. The scattering patterns sometimes contain sharp diffraction peaks that can be attributed to cuticular wax, which were removed using a simple rolling ball algorithm that excludes sharp features in the data.

### 5.4. Scattering intensity based sonograms

The sinograms, *I*(*x*, *R*_*y*_), were first calculated based on various features in the scattering profile, then converted to the corresponding tomograms (see below). The SAXS sinograms are based on the scattering intensity integrated in the *q* range of 0.1–0.15 Å^−1^. The cellulose and amorphous tomograms are based on intensities integrated within the *q* ranges of 1.55–1.63 and 1.28–1.37 Å^−1^, respectively. The CI tomograms were calculated from the cellulose and amorphous tomograms, based on the Segal definition:
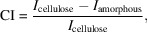
after normalization to account for the difference in the integrating ranges. Low-intensity voxels were excluded to avoid division-by-zero artefacts.

The data from the rice sample were processed differently due to the presence of starch. The intensity of the peaks attributed to starch was estimated using a rolling ball algorithm then integrated within the *q* ranges of 1.10–1.18 and 1.25–1.32 Å^−1^, where pure cellulose scattering does not show significant intensity (see Fig. S2). Similarly, the intensity for crystalline cellulose and amorphous components was integrated in the *q* ranges of 1.45–1.55 and 1.30–1.35 Å^−1^, which are minimally affected by the subtraction of the assumed starch scattering. While it is possible to fit each scattering profile based on a model that includes all know diffraction peaks from starch and cellulose, as well as amorphous scattering from other components, doing so would be very time consuming, given that each dataset contains ∼10^5^ individual scattering patterns.

### 5.5. Pre-scaling

The scattering intensity spans several orders of magnitude over the full *q* range of the data [see Fig. 1 ▸(*E*)]. Since the loss function in the decomposition algorithm is based on intensity, low-intensity features are more likely to be neglected. In order not to miss any important features, before decomposition we first multiplied the data with a shape factor (Fig. S3) that significantly reduces the dynamic range of the data without introducing any new features. In Figs. 3 ▸ and 4 ▸, the basis vectors are based on the modified data, while the recomposed data are divided by the same shape factor to recover the original dynamic range.

### 5.6. NMF

Decomposition by NMF was performed using *scikit-learn* (Pedregosa *et al.*, 2011 ▸), using the default Frobenius beta loss. The parameters were manually adjusted to obtain basis vectors that resemble realistic scattering profiles in Fig. 3 ▸. To estimate the number of components required to adequately represent the data, trial runs were performed with an increasing number of components, as shown in Fig. 3 ▸(*A*). The number of components is selected such that more components do not result in a significant decrease in the beta loss. For the analysis in Fig. 4 ▸, all default NMF parameters were kept intentionally, to produce a basis set that clearly does not represent physical components. In addition, due to the larger size of the dataset combined from eight samples, the randomized singular value decomposition (SVD) algorithm was used to estimate the required number of basis vectors, by placing the cut-off at ∼1% of the first SVD eigenvalue.

### 5.7. Tomographic reconstruction

The final virtual cross sections that correspond to each component were calculated from sinograms using standard tomography software, *tomopy* (Gürsoy *et al.*, 2014 ▸). The algorithm pml-hybrid (Chang *et al.*, 2004 ▸) was used with typically 100 iterations. The numerical values were normalized based on the values in the sinograms. Representative sinograms are shown in the supporting information. For consistency, all tomographic reconstructions for the same dataset were performed using the same rotation center value, which is determined from test-running reconstruction on the absorption data.

### 5.8. Accuracy of the tomograms

The accuracy of the reconstructed tomograms described above can be affected by several factors. First, the use of a finite number of components necessarily introduces some discrepancy between the actual dataset and the simplified set that is used in the subsequent data analysis. This can be evaluated by the relative error of NMF, calculated as the Frobenius beta loss normalized to the Frobenius norm of the original data and shown in Fig. 3 ▸(*A*). Some examples of the decomposition are also shown in Fig. S4. Second, the results produced by iterative reconstruction algorithms are not strictly mathematical inverse of the input sinograms. Combining the two steps together in future reconstruction algorithms may improve the accuracy of this analysis.

From the standpoint of scattering data collection, due to the finite sample size, different parts of the sample that contribute to the intensity in the same detector pixel in fact correspond to slightly different *q* values. In our measurements, the maximum lateral dimension of the sample is less than 5 mm, compared with the mean sample-to-detector distance of ∼350 mm. This corresponds to an uncertainty of ∼1.5% in *q*, or a smearing of ∼0.02 Å^−1^ at the location of the cellulose main peak. This is considered negligible, compared with the *q* grid of 0.01 Å^−1^ in the *q*–φ intensity map. Data collection can also benefit from better detector coverage in reciprocal space, as can be seen from Figs. 1 ▸(*C*) and 1 ▸(*D*).

### 5.9. Voxel size in the tomograms

This is set by the step size in the data collection, which is 5 µm for the results reported here and chosen to be close to the beam size. This is a good compromise for many plant samples. At this resolution, sufficient morphological details are preserved, allowing reasonable comparison with optical micrographs. With the current data-collection speed of 20 frames per second on the scattering detectors, which is limited by the speed of packaging the data into hdf5 files, data collection on a sample with a maximum lateral dimension of 3 mm takes 1 h. Given the same incident beam intensity, a smaller beam size would result in a higher rate of radiation deposited into the sample. This may require the data collection to run proportionally faster to limit radiation damage, resulting in lower scattering intensity. And it would take more data points to cover the same field of view. The compromise between sample morphology, data-collection speed, data quality and radiation damage ultimately determines the optimal voxel size in these measurements.

### 5.10. Clustering

After the tomographic reconstruction, we now have a set of distribution maps of the NMF components. In the parameter space, each voxel in the virtual cross section is represented by a point with coordinates corresponding to the amplitude of each NMF component. These voxels were grouped into clusters using the *k*-means algorithm implemented in *scikit-learn* (Pedregosa *et al.*, 2011 ▸), based on their locations in the parameter space. The number of clusters (*N*_c_) was selected based on the change in inertia, which is the objective function minimized by the *k*-means algorithm and defined as the sum of squared distances of data points to the cluster center, as the number of clusters was increased [the inset of Fig. 4 ▸(*C*)]. There is not a clear best choice. *N*_c_ = 3 was chosen to keep the maps from becoming too difficult to read. The coordinates of the centroid of each cluster were used to calculate the representative scattering intensity for each cluster, as shown in Fig. 4 ▸(*C*). Clustering analyses depend on the evaluation of distances in parameter space. Since we were interested in the material composition, this analysis was based on the relative magnitude of different components, after the length of all vectors that represent the data in the parameter space was normalized to unity. However, since the basis sets do not correspond to physical components, this may not be the best representation of material composition.

### 5.11. MFA decomposition

The angular intensity profile after removal of the estimated contribution from amorphous components is decomposed into intensity distributions that correspond to a set of discrete MFA values, from 0 to 90°, at an interval of 3° (Fig. S7), assuming an intrinsic peak width of 5°. The decomposition is performed using the non-negative least-square (NNLS) algorithm implemented in *scipy* (Virtanen *et al.*, 2020 ▸). This basis set is stored in a matrix *A*. To decompose the observed intensity *y*, we need to solve the equation *y* = *Ax*, where *x* gives the MFA distribution, by minimizing |*y* − *Ax*|^2^. To avoid over-fitting, two regularization terms are also added to simultaneously minimize the squared sum of the coefficients |*Ix*|^2^, where *I* is the identity matrix, and the difference between the neighboring terms |*Dx*|^2^, where the only non-zero elements in *D* are directly below the diagonal and have the value of −1. Effectively, the equation that the NNLS algorithm needs to solve becomes
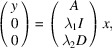
where the Lagrange multipliers λ_1_ and λ_2_ are adjusted manually to get the best results.

## 6. Concluding remarks

We have demonstrated scattering-based scanning tomography for plant samples, for which the rotational invariance of scattering intensity is generally satisfied when the growth direction is aligned to the rotation axis in the measurements. As an imaging method, scattering tomography provides direct visualization of the sample. At the same time, this method reveals information on the underlying structural information that is only accessible through analysis of the scattering intensity. The data-analysis workflow described in this article necessarily introduces some systematic errors. The approach of representing data as mathematical components enables the calculation of spatially resolved scattering intensity and further analysis using machine-learning methods. On the other hand, the fidelity of this component representation to the ground truth depends on the components chosen and the subsequent tomographic reconstruction. This is especially true for the inter-fibril correlation peak, whose position variation should require multiple components of different discrete positions to reproduce. Therefore, further work is needed to improve the accuracy of the data-analysis workflow. The rotational invariance is another issue, which is assumed but may not always be satisfied in the analysis of local MFAs. On the other hand, even as a qualitative diagnostic tool, scattering tomography as described here is still valuable for helping the experimenter to identify interesting areas in intact samples for more detailed studies.

Scattering-based imaging complements other imaging modalities that are based on absorption and fluorescence and therefore not sensitive to material structures. They can be particularly useful when used in combination. For instance, the variation in crystallinity or cellulose fibril correlation inferred from the SAXS intensity could be correlated with the distribution of chemical agents that are expected to break down cell-wall structures, to evaluate the efficacy of chemical treatment in bioenergy research. We are currently implementing simultaneous fluorescence and scattering data collection at the LiX beamline. The sample itself will absorb fluorescence emission from the interior of the sample, which can be corrected following recently developed methods (Ge *et al.*, 2022 ▸).

Radiation damage is an important consideration in synchrotron-based measurements on biological samples. In principle, the sample could be measured in a frozen-hydrated state, as is routinely done for protein crystallography and cryo-electron microscopy. However, performing flash freezing and maintaining the sample temperature during the measurement may not always be possible for larger samples. In the example presented here, all samples were measured dry, limiting the damage by radiation-induced free radicals. As a mitigation measure, we periodically translated the sample along the rotation axis during data collection to expose fresh parts of the sample to the X-rays and used the sinograms to monitor radiation damage as described earlier (an example of unmitigated radiation damage is shown in Fig. S9). Given that tomograms can be obtained even from scattering data of low intensities (Fig. S1), we should be able to further reduce radiation damage by shortening the exposure time for scattering-data collection. Future instrumentation developments to optimize data collection will be combined with refinement of tomographic reconstruction algorithms, to better account for the various assumptions we have made in the data-analysis workflow.

## Supplementary Material

Supporting figures. DOI: 10.1107/S1600577524004387/vl5025sup1.pdf

Supporting video. DOI: 10.1107/S1600577524004387/vl5025sup2.mp4
