# Supplementary material for: X-ray scattering based scanning tomography for imaging and structural characterization of cellulose in plants
Source: J Synchrotron Radiat. 2024 Jun 25;31(Pt 4):936–47. doi: 10.1107/S1600577524004387 (PMC11226170; doi:10.1107/S1600577524004387)
Supplement: Supplementary file 1 [file s-31-00936-sup1.pdf]

JOURNAL OF SYNCHROTRON RADIATION

Volume 31 (2024)

Supporting information for article:

X-ray scattering based scanning tomography for imaging and structural characterization of cellulose in plants

Lin Yang

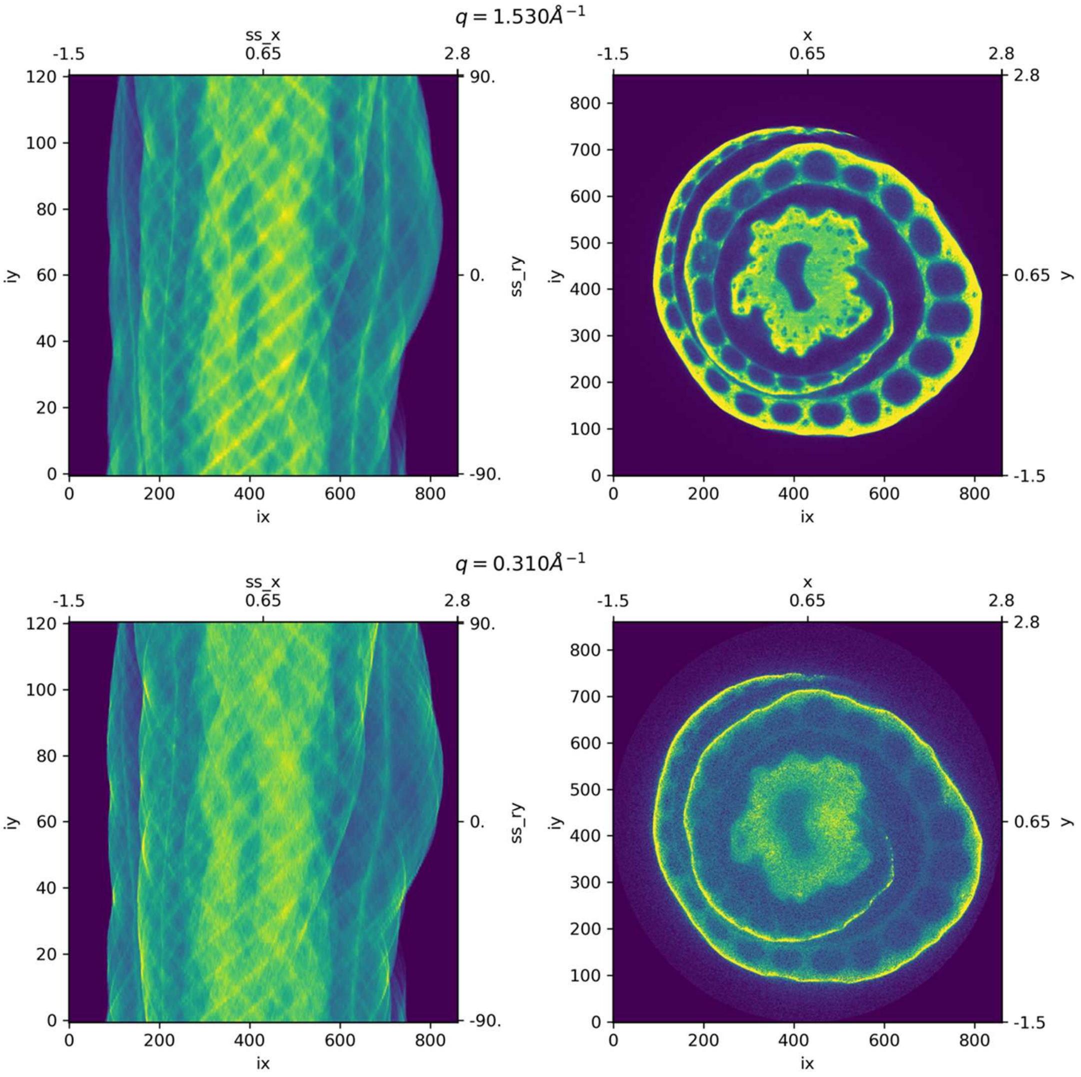

Figure S1 Examples of sinograms and tomographic reconstructions based on the scattering intensity from a single q value in the $I(q)$ profile: from the cellulose peak (top) and the region that show lower intensity and is free of any scattering features (bottom, q = 0.31 Å^{-1}). The color scale for the tomograms (right) has been set to intentionally emphasize low values. As expected, the quality of the bottom tomogram is clearly poorer, with ambiguous boundaries between plant materials and the empty space.

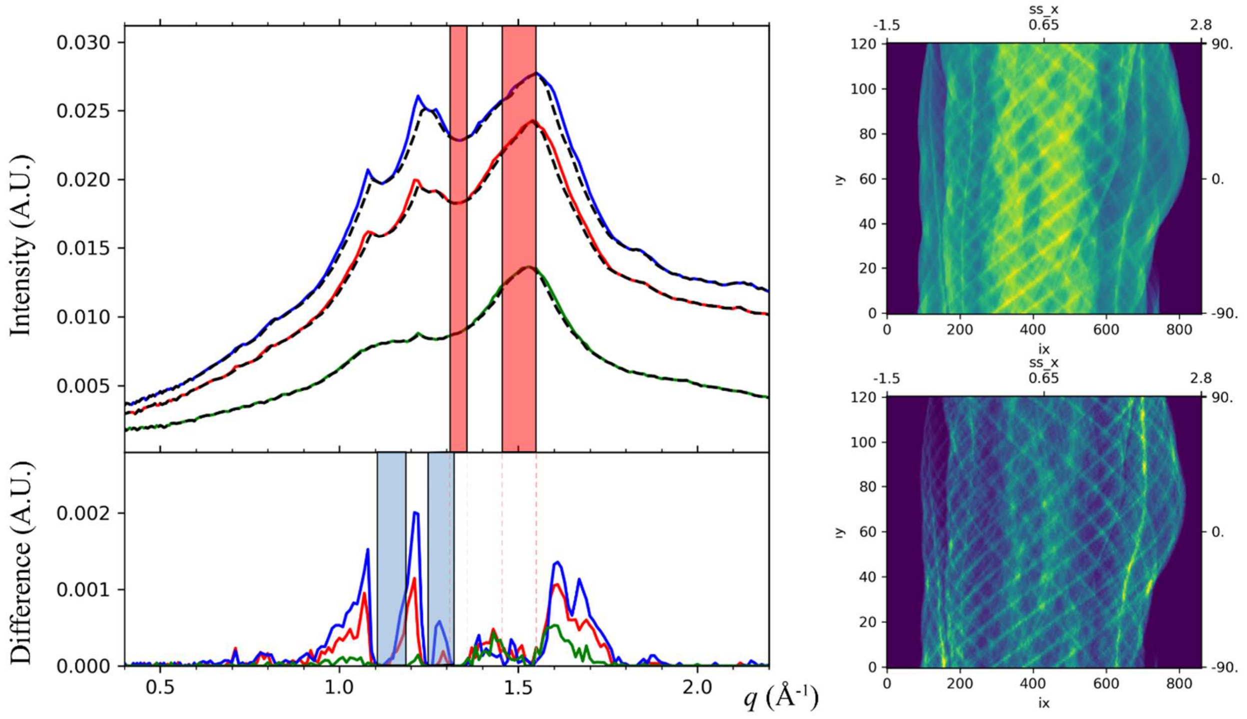

Figure S2 Estimate of starch and cellulose scattering intensity from the rice data. This process is demonstrated for 3 different scattering profiles (solid lines). The green line is chosen since it appears to contain only cellulose scattering. To extract the scattering intensity from starch, we first generate a smoothed intensity profile (black dashed lines) by applying the rolling ball algorithm. The difference between the original and smoothed profiles is used as a proxy for the (partial) starch scattering intensity (bottom panel). Clearly, this difference does not show correct peak shapes that are expected of starch scattering. However, within the *q*-ranges of 1.10-1.18 and 1.25-1.32 $Å^{-1}$ (blue boxes), the cellulose scattering contributes no intensity, therefore the intensity integrated should correctly reflect the abundance of starch. Conversely, within the *q*-ranges of 1.45-1.55 and 1.30-1.35 $Å^{-1}$ (red boxes), the original scattering intensity minimally deviates from the smoothed profile. Therefore, we use these intensity values to represent the abundance of cellulose and amorphous components. The resulting sinograms for cellulose and starch are shown on the right. The horizontal coordinates are sample translation (top, ss_x) and pixel index (bottom, ix). The vertical coordinates are the projection angle (right, ss_ry) and pixel index (left, iy).

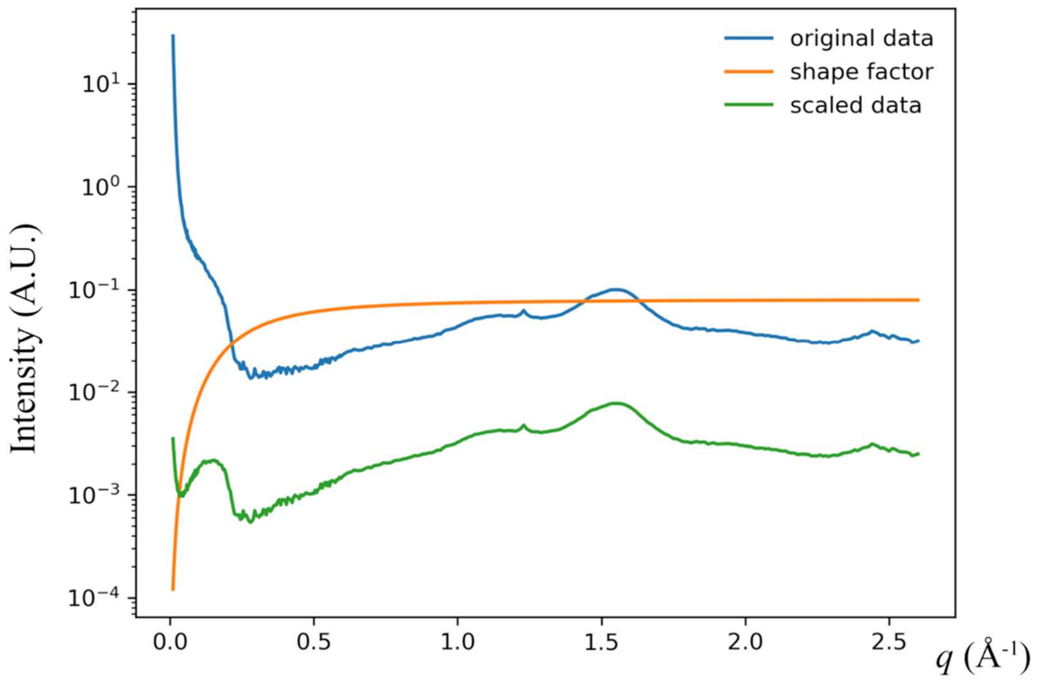

Figure S3 The shape factor is defined as $\frac{1}{q^{-a}+q_0{}^{-1}}$, using the values of a = 2 and q_0 = 0.08 Å^{-1}. An example of the data before and after shaping is shown below.

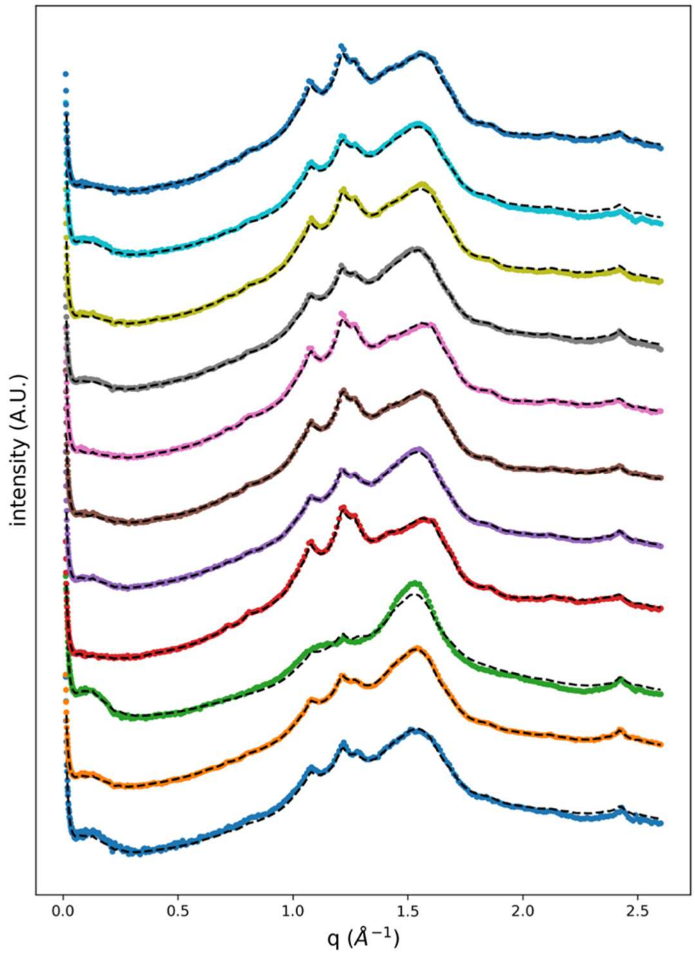

Figure S4 Examples of the NMF decomposition of the scattering data. The loss function, or the relative error cited in the text, from the NMF only provides an overall assessment of how well the components have captured the features in the data. Here are some examples of the initial scattering profiles (dots) and the decomposed results (dashed lines), randomly selected from the rice data shown in Figure 2.

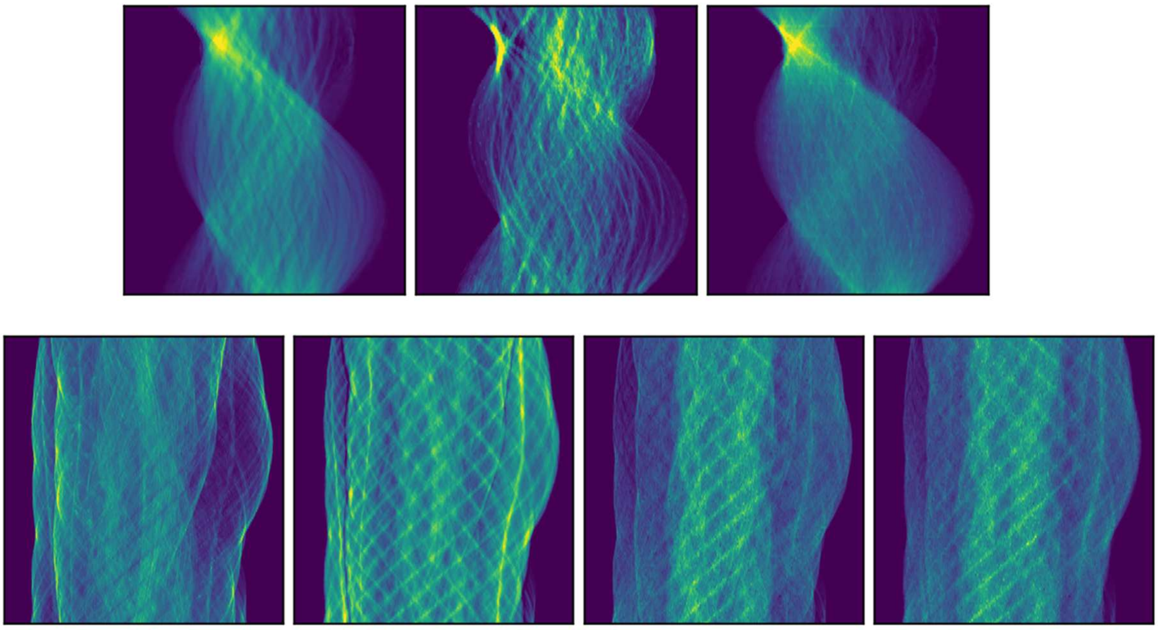

Figure S5 As examples, shown here are the sinograms that correspond to the basis vectors used for decomposing the bamboo and rice data in Figure 3. The coordinates are omitted for clarity. The angular range (vertical axis) is from -90 to 90 deg. The lateral position span is 2.1 mm for the bamboo data (top) and 4.3 mm for the rice data (bottom).

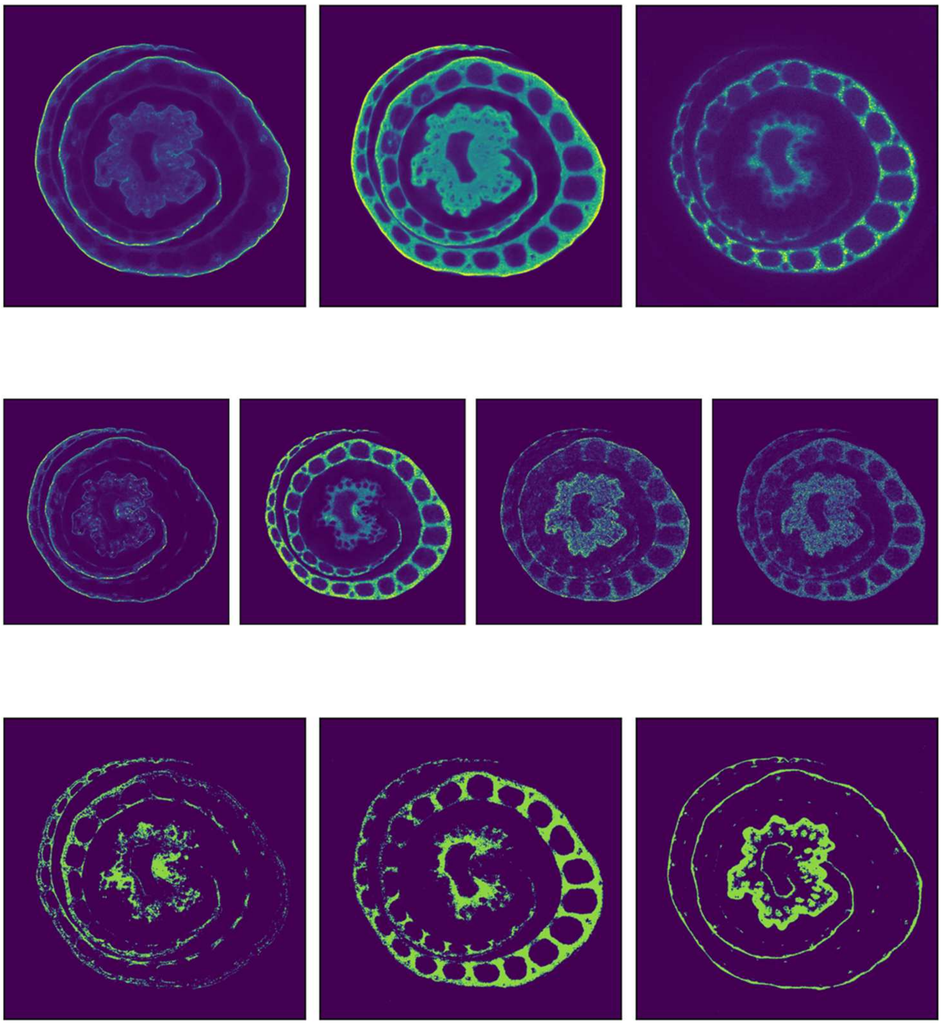

Figure S6 A comparison between tomograms obtained by different methods from the same sample: by features in the scattering intensity (top, same as Fig.2B, shown here are SAXS, cellulose, and starch), by components (middle, same as Fig.3E), and by cluster (bottom, same as Fig.4B-d).

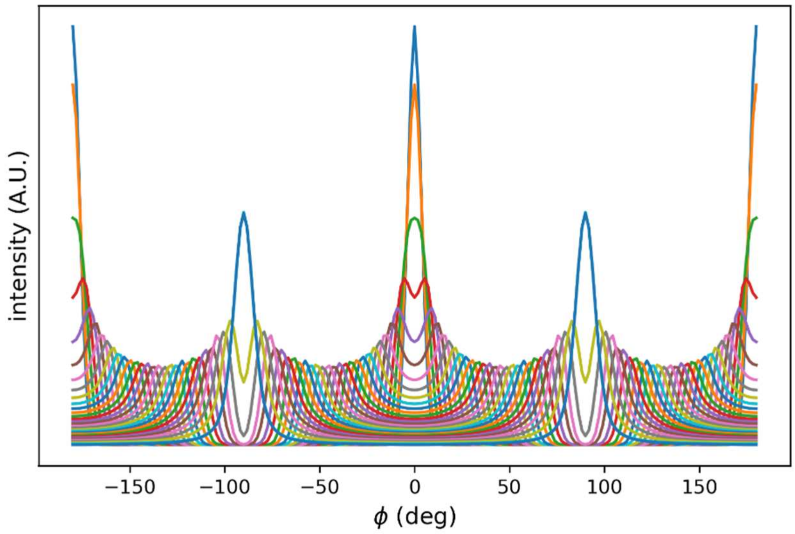

Figure S7 The basis set of azimuthal intensity profile used for calculating the MFA distribution. They are calculated for a set of predefined MFA values, ranging from 0 to 90 degrees at 3-degree intervals. An intrinsic peak width of 5 degrees is assumed.

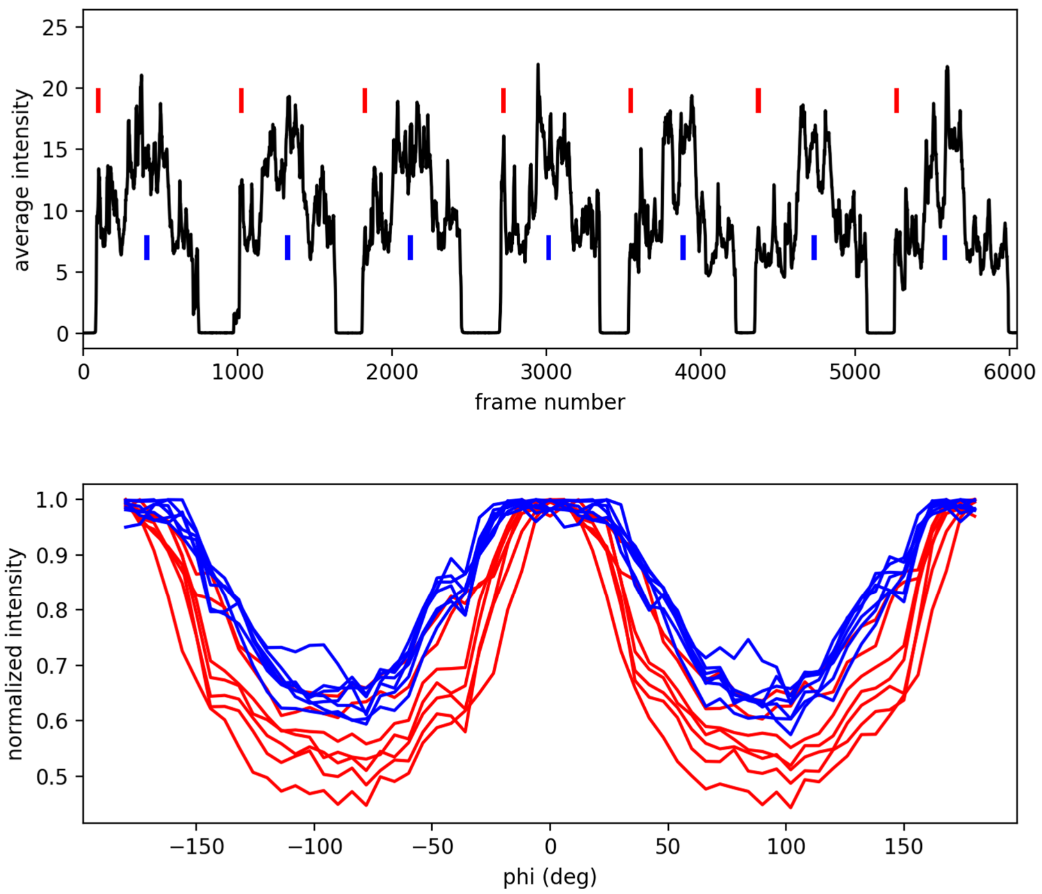

Figure S8 (top) The overall scattering intensity observed during the tomographic data collection on a rice sample. The blocks of non-zero intensity, corresponding to the x-scans of the beam across the sample, are separated by gaps of zero intensity, when the beam misses the sample. The red bars indicate positions when the beam illuminates the exterior of the rice sample, while the blue bars are locations where the beam passes through the center of the sample. (bottom) The azimuthal intensity profiles (normalized to the maximum intensity) observed at these locations are plotted using the corresponding colors. The red curves consistently show narrower angular distributions.

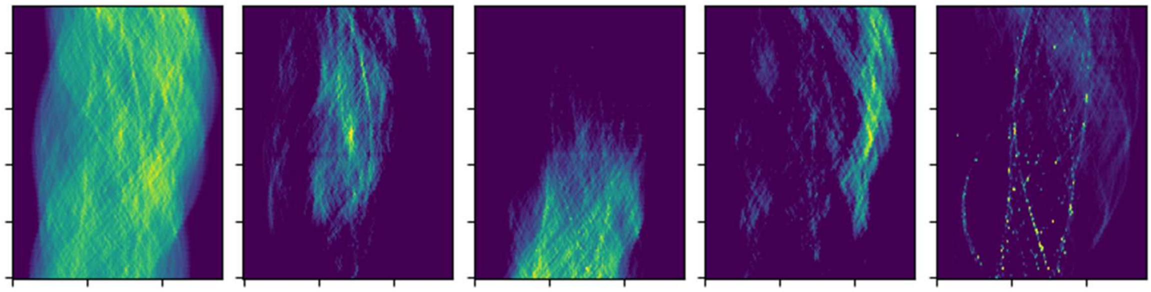

Figure S9 An example of radiation damage observed in earlier experiments, when projections (x-scans) were collected at the same location along the growth direction and monotonically increasing projection angles. The data are shown as sinograms, corresponding to components obtained using singular value decomposition (SVD). The time-dependence (vertical axis) of the intensity in the middle three components is a clear indication of the sample structure changing, presumably as the consequence of radiation damage. This change is no longer observed once the data collection strategy of shifting samples slightly along the growth direction is adopted.
